# Supplementary material for: Mechanisms governing the pioneering and redistribution capabilities of the non-classical pioneer PU.1
Source: Nat Commun. 2020 Jan 21;11:402. doi: 10.1038/s41467-019-13960-2 (PMC6972792; doi:10.1038/s41467-019-13960-2)
Supplement: Supplementary file 7 — Source data [file 41467_2019_13960_MOESM7_ESM.zip › Source_Data/FigureS2/FigureS2B_MotifScanOutput/KmeansCluster3/homerResults/motif18.similar.html]

motif18

## Information for motif18

G
T
C
A
G
T
C
A
A
G
T
C
G
A
C
T
C
A
G
T
C
T
G
A
T
A
G
C
G
C
T
A
T
C
G
A
C
A
G
T
A
G
T
C
T
G
C
A
  
Reverse Opposite:  

A
C
G
T
T
C
A
G
G
T
C
A
A
G
C
T
C
G
A
T
A
T
C
G
G
A
C
T
G
T
C
A
C
T
G
A
T
C
A
G
C
A
G
T
C
A
G
T
  

|  |  |
| --- | --- |
| p-value: | 1e-53 |
| log p-value: | -1.241e+02 |
| Information Content per bp: | 1.526 |
| Number of Target Sequences with motif | 111.0 |
| Percentage of Target Sequences with motif | 2.33% |
| Number of Background Sequences with motif | 98.2 |
| Percentage of Background Sequences with motif | 0.23% |
| Average Position of motif in Targets | 94.3 +/- 70.7bp |
| Average Position of motif in Background | 102.3 +/- 69.9bp |
| Strand Bias (log2 ratio + to - strand density) | 0.3 |
| Multiplicity (# of sites on avg that occur together) | 1.00 |
| Motif File: | file (matrix) reverse opposite |

### Similar de novo motifs found

|  |  |  |  |  |  |  |  |
| --- | --- | --- | --- | --- | --- | --- | --- |
| Rank | Match Score | Redundant Motif | P-value | log P-value | % of Targets | % of Background | Motif file |
| 1 | 0.940 | T C G A C T G A G T A C A G C T C G A T C T G A A G T C G T C A T C G A C G A T A G T C C T G A G A C T | 1e-53 | -123.209113 | 2.62% | 0.33% | motif file (matrix) |
| 2 | 0.894 | C G T A C G T A A G T C A G C T C A G T T C G A A G T C G T C A T C G A C A G T A G T C T G C A A C G T C T A G | 1e-52 | -120.231686 | 2.52% | 0.31% | motif file (matrix) |
| 3 | 0.923 | G C T A G T C A A G T C A G C T C G A T C T G A A T G C G C T A C T G A A C G T A G T C | 1e-48 | -110.790940 | 2.47% | 0.33% | motif file (matrix) |
| 4 | 0.780 | C G T A A G T C A C G T A G C T C G T A A G T C C G T A C T A G A C G T A G T C G C T A C G A T A C T G | 1e-41 | -95.638728 | 1.97% | 0.24% | motif file (matrix) |
